# Impact of pigment epithelium-derived factor on colorectal cancer *in vitro* and *in vivo*

**DOI:** 10.18632/oncotarget.24953

**Published:** 2018-04-10

**Authors:** Rhiannon L. Harries, Sioned Owen, Fiona Ruge, Meleri Morgan, Jun Li, Zhangtao Zhang, Keith G. Harding, Jared Torkington, Wen G. Jiang, Jun Cai

**Affiliations:** ^1^ Cardiff–China Medical Research Collaborative, Cardiff University School of Medicine, Heath Park, Cardiff, UK; ^2^ University Hospital of Wales, Heath Park, Cardiff, UK; ^3^ Department of General Surgery, Beijing Key Laboratory of Cancer Invasion and Metastasis Research and National Clinical Research Center for Digestive Diseases, Beijing Friendship Hospital, Capital Medical University, Beijing, China; ^4^ Welsh Wound Innovation Centre, Llantrisant, UK

**Keywords:** pigment epithelial derived factor, PEDF, colorectal cancer, metastases

## Abstract

Pigment epithelial derived factor (PEDF) is a secreted glycoprotein that is a non-inhibitory member of the serine protease inhibitor (serpin) family. PEDF exhibits multiple biological properties including neuroprotective, anti-angiogenic, and immune-modulating. Interestingly, PEDF exerts the inhibitory effects in cancers derived from certain tissues, including prostatic, ovarian, and pancreatic carcinomas. The current study aimed to elucidate its role in colorectal cancer development. PEDF expression in human colorectal cancer tissue was assessed using quantitative polymerase chain reaction (qPCR) and immunohistochemical staining (IHC). The effect of treatment with recombinant PEDF on cellular function was examined using *in vitro* functional assays. PEDF expression was downregulated in colorectal cancer cell tissue. Treatment with recombinant PEDF resulted in significant decreases in the rate of colorectal cancer cell migration and invasion and an increase in cellular adhesion in colorectal cancer cell lines examined. These results indicate that upregulation of PEDF expression may serve as a new strategy for further investigation of therapeutic relevance to the prevention of the metastatic spread of colorectal cancer.

## INTRODUCTION

Pigment epithelium-derived factor (PEDF), also known as early population double level cDNA-1 (EPC1), is a 50 kDa secreted glycoprotein. It was first identified when Tombran-Tink’s group were studying human retinal cell development. They found a factor that was secreted by the human foetal retinal pigment epithelial cells and showed it to be a potent neurite promoting factor [1]. Subsequently, PEDF was found to be a member of the non-inhibitory serpin gene family [2]. The gene encoding PEDF (SERPINF1) is localised to the chromosome 17p13.1 [3]. There is evidence that PEDF is pleiotropic with multiple biological properties including neuroprotective, anti-tumorigenic and immune-modulating [4–5] and has been shown to be one of the most potent endogenous inhibitors of angiogenesis, more so than angiostatin, endostatin, and thrombospondin-1 [6–7].

PEDF exerts anti-angiogenic activities by arresting endothelial cell proliferation and migration, an activity which has been shown to occur even in the presence of vascular endothelial growth factor (VEGF) [6, 8–9]. The mechanisms of action appear to be multifactorial; suggested underlying mechanisms of PEDF biological effects on endothelial cells involve a complex cross-talk between the signal events triggered by both pro-angiogenic and anti-angiogenic molecules [10].

PEDF expression has been found to be lower in solid tumour tissue when compared to normal tissue from the same organ [11–16], suggesting that loss of PEDF expression may play a crucial role in tumorigenesis. Previous studies have also demonstrated that PEDF expression is downregulated with worsening prognostic factors in a range of cancers [11–12, 15, 17–20] and that treatment with recombinant PEDF has shown some benefit in cellular functional models [14, 19, 21–27], likely due, in part, to PEDF inhibiting aberrant angiogenesis, which leads to normalisation of healthy vasculature.

However, the evidence investigating the role of PEDF in colorectal cell line function has been limited. The current study examined the expression of PEDF in colorectal cancer tissue and its effect on *in vitro* cellular function of colorectal cancer cells.

## RESULTS

### Low expression of PEDF mRNA in colorectal cancer tissues

**Figure 1:**
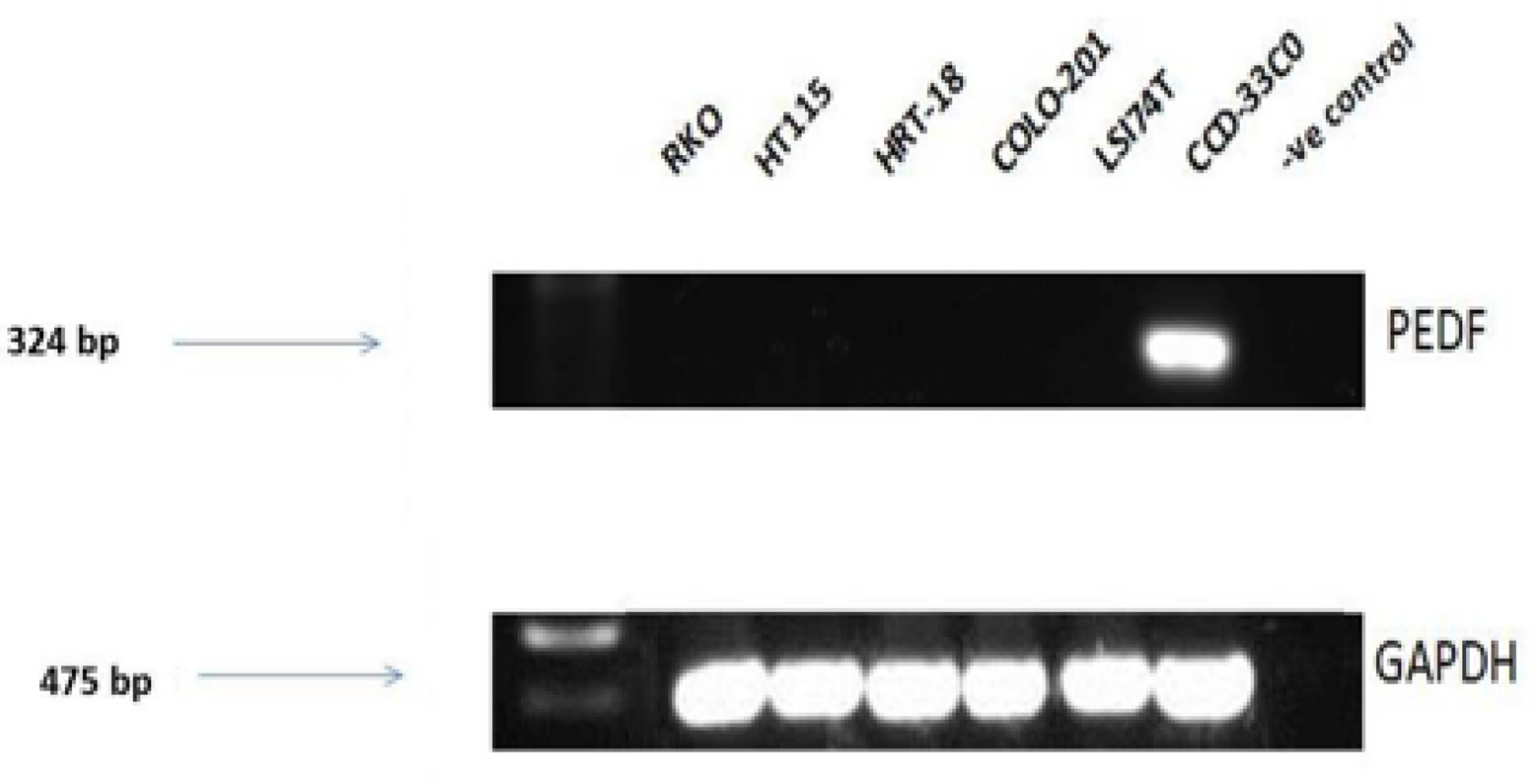
Transcript expression levels in PEDF in colorectal cell lines Control = Nuclease free water and all gels were run with a molecular weight marker used to identify band sizes.

**Figure 2:**
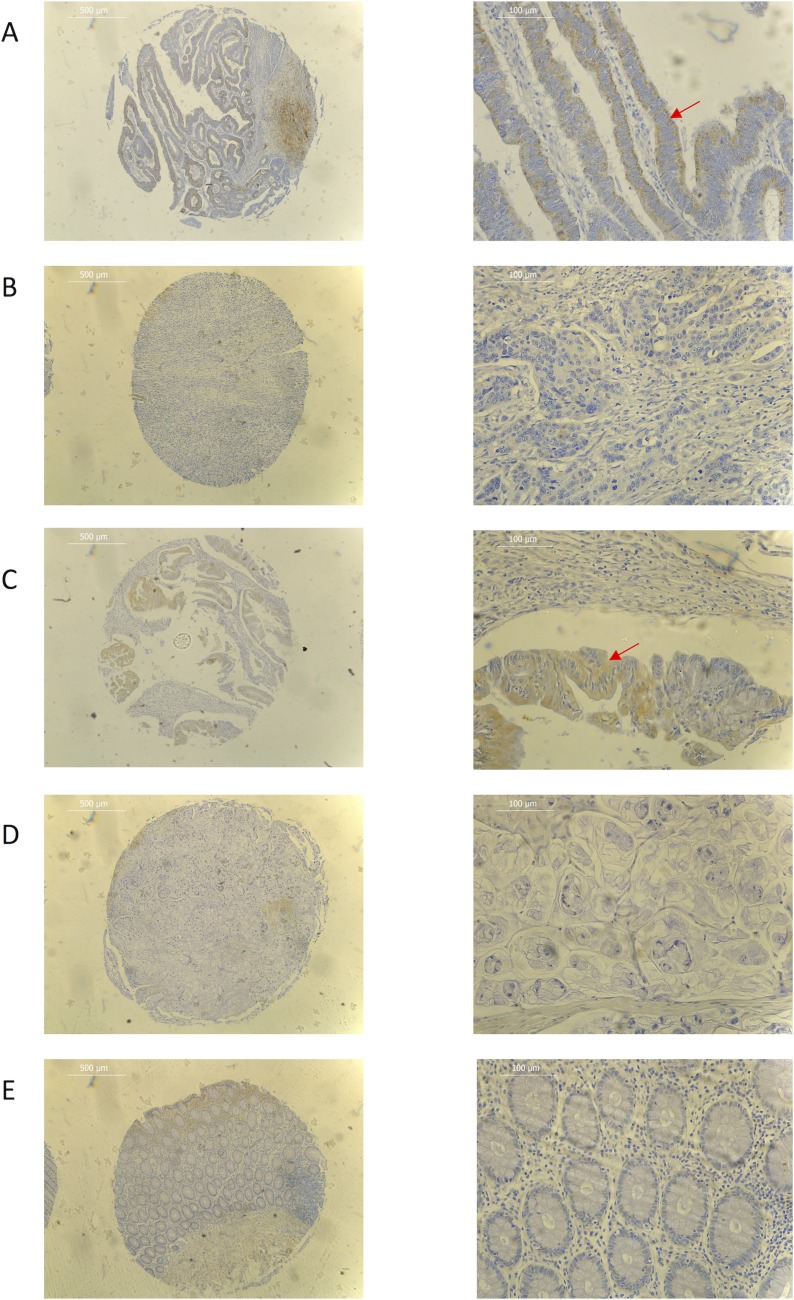
Representative immunohistochemistry images for (**A**) well differentiated adenocarcinoma (**B**) poorly differentiated adenocarcinoma (**C**) well differentiated mucinous adenocarcinoma (**D**) poorly differentiated mucinous adenocarcinoma (**E**) normal colorectal tissue samples. Red arrow shows cytoplasmic tumour staining. ×40 (L) and ×200 (R) magnification used. Scale bar represents 500 µm (L) and 100 µm (R).

Expression screening for PEDF was performed using both colorectal cancer tissue samples obtained from the clinical cohort and colorectal cancer cell lines. PEDF expression was lower in all the colorectal cancer cell lines when compared to the CCD-33C0 colorectal fibroblast cell line, used as a positive control (Figure 1**)**. On transcript analysis, mRNA expression of PEDF was lower in colorectal tumour tissue when compared to matched normal colorectal tissue from colorectal cancer patients (Table 1**)**. On IHC staining highest expression of PEDF was seen within smooth muscle, endothelial cells and fibroblasts (Figure 2**)**. There was some slight cytoplasmic staining seen within cancer adjacent and normal colorectal tissue. However, there was reduced expression overall.

**Table 1:** Correlation between PEDF expression and clinical parameters in colorectal cohort

	*N*	Median transcript copy number	IQR	*P* value
Tumour	406	3.64 × 10^–5^	1.05 × 10^−9^–4.40 × 10^−3^	
Normal matched tissue	209	1.05 × 10^–3^	6.84 × 10^−9^–4.18 × 10^−2^	<0.001^*^
Female	185	1.54 × 10^–4^	3.95 × 10^−8–^1.02 × 10^−2^	
Male	221	1.51 × 10^–5^	2.52 × 10^−10–^2.36 × 10^−3^	0.01^*^
Aged 64 years or younger	163	8.78 × 10^–6^	1.17 × 10^−12^−3.46 × 10^−3^	
Aged 65 years or older	184	1.04 × 10^–6^	1.17 × 10^−12^−1.36 × 10^−3^	0.645
Female aged 64 years or younger	75	1.08 × 10^–5^	1.17 × 10^−12^− 4.18 × 10^−3^	
Male aged 64 years or younger	88	4.39 × 10^–6^	2.45 × 10^−12^−1.44 × 10^−3^	0.729
Female aged 65 years or older	84	1.47 × 10^–5^	1.17 × 10^12^–2.29 × 10^−3^	
Male aged 65 years or older	100	5.63 × 10^–7^	1.17 × 10^−12^–7.25 × 10^−4^	0.107
Smoker	95	1.78 × 10^–5^	8.34 × 10^−11^–3.02 × 10^−3^	
Non-smoker	239	6.44 × 10^–5^	1.31 × 10^−10^–3.92 × 10^7^	0.70
History of other cancers	17	4.02 × 10^–5^	6.54 × 10^−7^–2.79 × 10^−3^	
No history of other cancers	375	5.32 × 10^–5^	9.62 × 10^−10^–4.50 × 10^−3^	0.617
Family history of colorectal cancer	47	1.85 × 10^–5^	1.69 × 10^−1 0^–1.15 × 10^−3^	
No family history of colorectal cancer	344	6.93 × 10^–5^	4.09 × 10^−9^–5.25 × 10^−3^	0.382
Tumour location				
Colon	263	2.91 × 10^–6^	5.00 × 10^−14^–4.78 × 10^−3^	
Rectum	143	1.25 × 10^–4^	4.44 × 10^−6^–3.40 × 10^−3^	<0.001^*^
Tumour differentiation				
Well differentiation	84	1.31 × 10^–4^	3.36 × 10^−7^–4.00 × 10^−3^	
Moderately differentiation	231	2.49 × 10^–5^	1.97 × 10^−10^–3.31 × 10^−3^	
Poorly differentiation	37	9.69 × 10^–7^	1.31 × 10^−11^–1.83 × 10^−3^	0.187
Tumour type				
Adenocarcinoma	307	6.44 × 10^–5^	9.62 × 10^−10^–3.67 × 10^−3^	
Mucinous adenocarcinoma	49	4.92 × 10^–5^	4.45 × 10^−10^–2.41 × 10^−3^	0.98
Duke’s stage				
A	22	1.94 × 10^–4^	3.17 × 10^−6^–5.03 × 10^−3^	
B	170	1.63 × 10^–5^	4.78 × 10^−11^–3.33 × 10^−3^	
C	156	1.29 × 10^–4^	2.45 × 10^−8^–6.66 × 10^−3^	
D	30	5.12 × 10^–5^	2.24 × 10^−9^–2.25 × 10^−3^	0.16
Pathological T stage				
T1	0			
T2	34	1.65 × 10^–4^	3.09 × 10^−7^–4.37 × 10^−3^	
T3	201	2.66 × 10^–5^	3.55 × 10^−10^–2.51 × 10^−3^	
T4	148	5.78 × 10^–5^	1.50 × 10^−10^–7.76 × 10^−3^	0.45
No nodal involvement	201	2.77 × 10^–5^	2.25 × 10^−10^–3.46 × 10^−3^	
Nodal involvement	181	7.30 × 10^–5^	7.21 × 10^−9^–5.33 × 10^−3^	0.23
No metastatic disease	273	6.04 × 10^–5^	9.42 × 10^−11^–3.69 × 10^−3^	
Metastatic disease	31	1.45 × 10^–5^	1.09 × 10^−9^–2.11 × 10^−3^	0.54
Radical surgery	341	6.48 × 10^–5^	4.09 × 10^−9^–4.42 × 10^−3^	
Palliative surgery	45	1.51 × 10^–5^	1.50 × 10^−12^–5.33 × 10^−3^	0.54

^*^p ≤ 0.05.

### Association of PEDF transcript levels with clinical and histopathological features of colorectal cancer

On transcript analysis, PEDF was more highly expressed in females with colorectal cancer within this cohort when compared to males with colorectal cancer (*p* = 0.01), and in rectal tumours compared with colonic tumours (*p* < 0.001) (Table 1). Whilst there was an obvious decline in mRNA expression of PEDF with worsening tumour grade, this trend was not found to be significant (*p* = 0.187). No other demographic or clinicopathological association were found to be statistically significant. Unfortunately, survival data was not available due to the short follow-up period of this cohort.

On IHC, tumour expression of PEDF was more pronounced in well-differentiated mucinous adenocarcinomas when compared to poorly differentiated mucinous adenocarcinomas and all grades of adenocarcinoma (Figure 2). There was a significant decrease in expression with worsening tumour grade in both adenocarcinomas and mucinous adenocarcinomas (*p* = 0.008 and *p* < 0.001, respectively), while there was no difference seen in expression in tumour location (colon vs. rectum), Dukes Stage or TNM Stage.

### Effect of PEDF on cellular function in colorectal cancer cells

**Figure 3:**
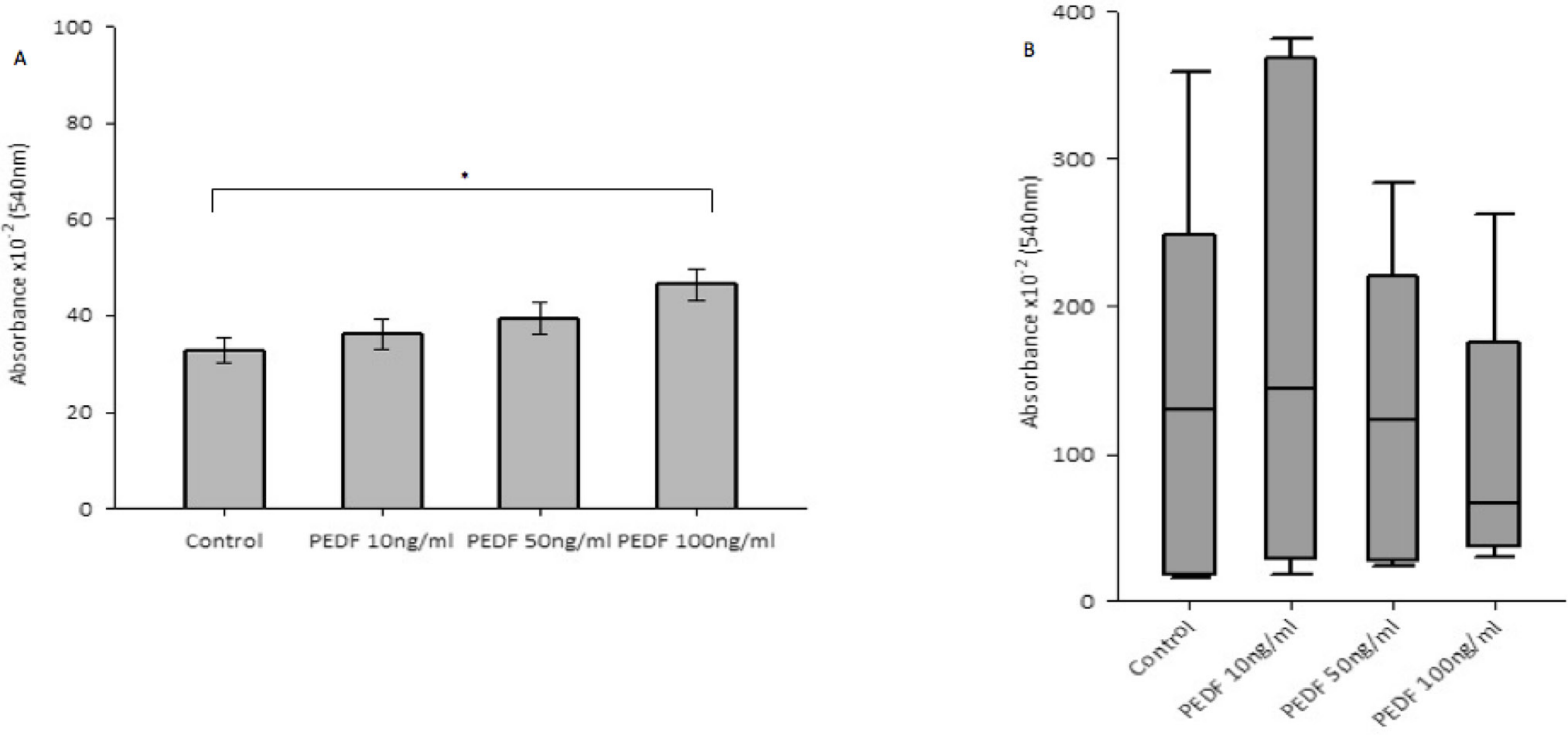
**(A**) Impact of rhPEDF on cellular attachment in HT115 cells. Mean values of 3 independent repeats are shown. Error bars represent SEM. (**B**) Impact of rhPEDF on cellular attachment in HRT-18 cells. Median values and IQR of 3 independent repeats are shown. Error bars represent 95% confidence intervals. Absorbance × 10^–2^ (540 nm) readings used as a marker of cellular attachment, in response to varying concentrations of rhPEDF. ^*^p < 0.05.

**Figure 4:**
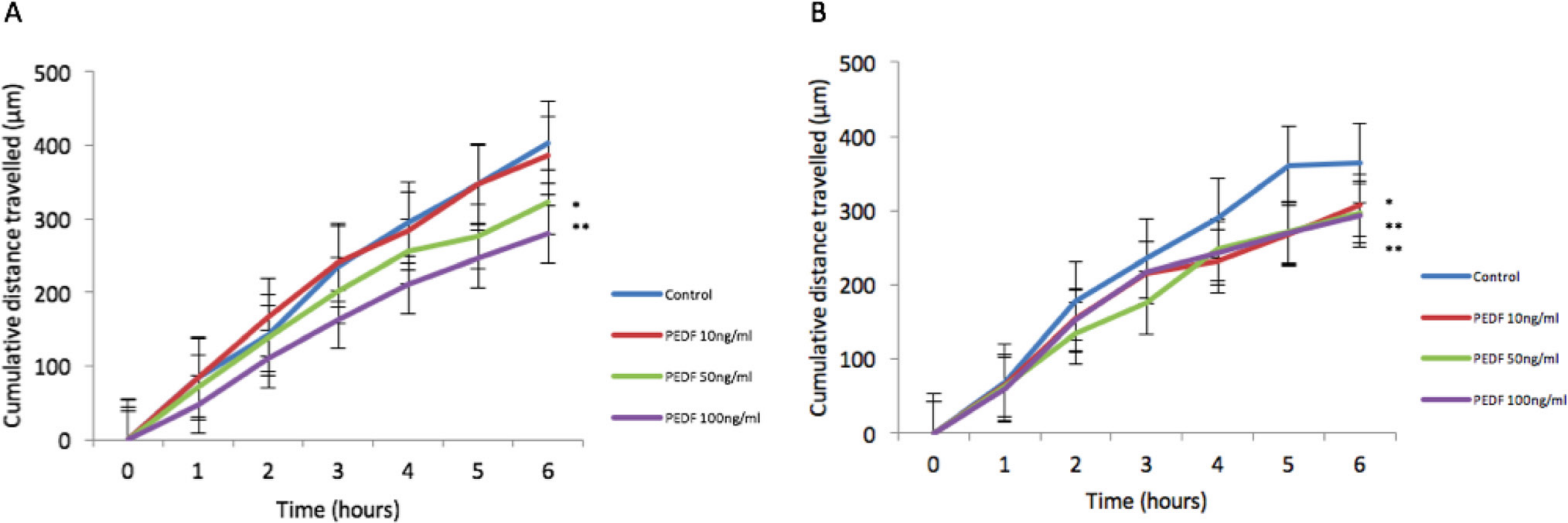
**(A**) Impact of rhPEDF on HT115 cellular migration assessed through scratch migration assay. (**B**) Impact of rhPEDF on HRT-18 cellular migration assessed through scratch migration assay. Cumulative distance travelled following scratch is shown and taken as representative of migration in cells compared to control and varying doses of rhPEDF. Mean values of 3 independent repeats are shown. Error bars represent SEM. ^*^p < 0.05 ^**^p < 0.001.

There was no statistically significant difference seen in both cellular growth and invasion in either HT115 or HRT-18 cell line comparing control in the presence of varying concentrations of rhPEDF. There was a statistically significant increase in the attachment of HT115 cells with the treatment of rhPEDF (100 ng/ml) when compared to the control (*p* = 0.003). However, this significant increase was not observed with 10 ng/ml or 50 ng/ml treatment doses of rhPEDF (Figure 3). No difference was demonstrated in the attachment of HRT-18 cells with the treatment of rhPEDF compared to the control. There was a statistically significant decrease in HT115 migration rate, evident for both 50 ng/ml and 100 ng/ml rhPEDF treatment doses (*p* = 0.007 and *p* < 0.001, respectively), when compared to untreated HT115 cells and 10 ng/ml rhPEDF treatment dose (Figure 4). A similar effect was demonstrated in the HRT-18 cell line; a statistically significant decrease in HRT-18 migration rate, was evident for all doses of rhPEDF compared to untreated HRT-18 cells. (rhPEDF 10 ng/ml vs control *p* = 0.002, rhPEDF 50 ng/ml *p* < 0.001, rhPEDF 100 ng/ml *p* < 0.001) (Figure 4).

## DISCUSSION

The results of this study demonstrate that PEDF mRNA expression was lower in colorectal cancer cell lines when compared to normal colorectal fibroblasts and in colorectal cancer tissue compared to normal tissue. These findings support those of Wågsäter *et al.* (2010) and Ji *et al.* (2013), who reported a significant reduction in serum PEDF levels in colorectal cancer patients compared to healthy controls [28–29].

Interestingly, mRNA expression of PEDF was observed to be higher in female colorectal cancer patients when compared to male colorectal cancer patients. This is contrary to both Yamagishi *et al.* (2006) and Wågsäter *et al.* (2010) who established that plasma samples from healthy male controls had higher levels of PEDF when compared to healthy female controls [28, 30], however a similar finding in colorectal cancer patients was either not assessed or demonstrated, in the respective studies. Other studies evaluating PEDF expression in other solid tumours did not display significance difference between genders [18, 31]. There is evidence to support a survival advantage in younger females compared to younger males, but with an opposite pattern in older patients [32–34]. One of the main reasons suggested for this effect is the favourable effect of endogenous female sex hormones [35]. However, it may be plausible that the gender difference in expression of PEDF may be responsible for the survival difference seen. In our cohort, we found no difference in PEDF expression comparing both genders in age groups 64 years or less and 65 years and over (*p* = 0.729 and *p* = 0.107, respectively).

Rectal tumour tissue appeared to express higher mRNA levels of PEDF when compared to colonic tumour tissue. Díaz *et al.* (2008) detected a similar difference with higher mRNA expression of PEDF in tissue from rectal tumours when compared to tissue from colonic tumour [36]. Cai *et al.* (2006b) found conflicting results on a cellular level with stronger PEDF expression in HT115 colonic adenocarcinoma cell line and weaker expression in HRT-18 rectal adenocarcinoma cell line [11], however our results showed little difference in the expression of these two cell lines and therefore there may be other factors in these cell lines that may be responsible for the expression levels shown, such as such as tumour grading or stage.

Mucinous adenocarcinoma tissue showed more positive tumour expression when compared to adenocarcinoma tissue on immunohistochemical staining, which is consistent with work from Ji *et al.* (2013) where patients with mucinous adenocarcinoma displayed higher PEDF plasma levels than adenocarcinoma patients [29]. This is perhaps a slightly surprising result, as mucinous adenocarcinoma of the colon or rectum is well recognised to have a poorer survival rate when compared to non-mucinous adenocarcinoma [37] and hence there must be other influences, yet to be identified, that are specific to mucin-producing tumours that are responsible for the higher expression levels of PEDF.

The most frequent cause of mortality from colorectal cancer is related to the effects of metastatic spread, and therefore treatments aimed at its prevention may prove highly beneficial for the long-term survival of colorectal cancer patients. Tumour metastasis depends on several cancer cell characteristics including cellular proliferation, adhesion, invasion and migration, and hence the capability to adapt to *in vivo* environments and outcompete with normal cells for resources necessary for survival. To date, the exact mechanisms of PEDF on colorectal cancer metastases remain poorly understood.

In this study, treatment with rhPEDF appeared to significantly decrease the rate of cellular migration. To date, the effect of rhPEDF on cellular function has not been studied in colorectal cell lines, but has been reported in a number of other solid tumours. Hong *et al.* (2014) reported a significant decrease in both breast cancer cell migration and invasion, and found that PEDF inhibited breast cancer cell migration and invasion by down-regulating fibronectin and subsequent MMP2/MMP9 reduction via p-ERK and p-AKT signalling pathways [22]. Correspondingly, Tan *et al.* (2010) found a 20% decrease in cellular invasion in PEDF treated chondrosarcoma cell when compared to control cells [27]. These findings support our results presented in this study.

In the adhesion studies presented in this study, treatment with rhPEDF resulted in an increase in cellular attachment. Tan *et al.* (2010) found a similar finding when examining chondrosarcoma cells [27]. Increased adhesion certainly presents a beneficial effect in the prevention of metastatic progression, by contributing to a decreased ability for the cancer cells to invade and migrate.

In conclusion, our results confirmed that PEDF expression was higher in normal colorectal tissue and cells compared to cancer tissue and cells and that PEDF administration confers an inhibitory effect on the migration and invasion of colorectal cancer cells. Future studies should examine the possible reasons behind a difference in PEDF expression patterns between the genders and tumour location and assess cellular function on a range of colorectal cancer cell lines (including metastatic and mucinous adenocarcinoma) and to explore the effect of cellular function in *in vivo* models using colorectal tumour xenografts within animal models.

## MATERIALS AND METHODS

### Cell culture

Human colorectal cancer cell lines RKO, COLO-201, LSI74T and colorectal fibroblast cell line CCD-33C0 were purchased from American Type Culture Collection (ATCC) (Rockville, Maryland, USA). Human colorectal cancer cell lines HT115, HRT-18 were purchased from Sigma-Aldrich (Poole, Dorset, UK). The cells were maintained in Dulbecco’s modified Eagle’s medium (DMEM)-F12 medium supplemented with 10% foetal calf serum and antibiotics. Polyclonal rabbit anti-PEDF was obtained from Santa Cruz Biotechnology Inc. (Santa Cruz, Texas, USA). Recombinant PEDF (rhPEDF) was purchased from R&D Systems Europe Ltd (Abingdon, Oxfordshire, UK) and cellular functional assays were performed at doses of 10 ng/ml, 50 ng/ml and 100 ng/ml versus control medium. HT115 and HRT-18 were used as representative colorectal cancer cell lines for cellular functional assays. Unless otherwise stated, other materials and reagents were purchased from Sigma-Aldrich Ltd (Poole, Dorset, UK).

### Collection of colorectal tissue samples

Colorectal cancer tissues (*n* = 406) and normal matched colorectal tissue (*n* = 209) were collected during surgery, and stored at −80° C until use and used for transcript analysis. Histopathological details were obtained from pathological reports. Ethical approval was obtained from Beijing Friendship Hospital, China and consent received from all patients included in the cohort. Colonic cancer tissue microarray (T054b) and rectal cancer tissue microarray (RE961) purchased from US Biomax Inc. (Rockville, USA) were used for immunohistochemical staining, and these microarrays included normal tissue and cancer adjacent normal tissue. All specimens were registered and stored according to Human Tissue Act regulations.

### RNA extraction, reverse transcription polymerase chain reaction (RT-PCR) and quantitative PCR

RNA isolation was performed using TRI reagent^®^. RNA concentration was determined using a UV Spectrophotometer (WPA UV 1101: Biotech Photometer, Cambridge, UK). Reverse transcription was performed using GoTaq Green master mix (Promega, Madison, USA) with 500ng of RNA with each RT reaction. The quality of cDNA was verified by examining a housekeeping gene, glyceraldehyde 3-phosphate dehydrogenase (GAPDH). Conventional PCR was carried out using primers for PEDF (Table 2). The conditions for PCR were: 94° C for 5 minutes, then 34 cycles of 94° C for 30 seconds, 55° C for 30 seconds, 72° C for 40 seconds, followed by a final extension at 72° C for 10 minutes. Visualisation of products took place on 0.8% agarose gel using SYBR safe DNA gel stain (Invitrogen, Manchester, UK). PEDF transcript levels in the colorectal tissue specimens were determined using real-time qPCR, using Amplifluor™ technology as described before [37]. In brief, the ampliflour probe consists of a 3′ region specific to the Z-sequence (ACTGAACCTGACCGTACA) present on the target specific primers and a 5′ hairpin structure labelled with a flourophore [38]. The flourophore hairpin structure is linked to an acceptor moiety and therefore acts as a fluorescence quencher preventing any signal from being detected. During the qPCR reaction, the uniprobe (Millipore, Watford, UK) becomes incorporated and acts as a template for DNA polymerisation, in which DNA polymerase uses its 5′-3′ exonuclease activity to degrade and unfold the hairpin structure. This disrupts the energy transfer between the quencher and flourophore and results in sufficient fluorescence to be emitted and detected. The fluorescent signal emitted during each cycle is directly correlated to the amount of DNA that has been amplified. Pairs of qPCR primers were designed using Beacon Designer^™^ software (PREMIER Biosoft, Palo Alto, California, USA) as discussed in Table 2, but with the additional Z sequence added to the antisense primer, complimentary to the universal Z probe (Intergen Inc., Oxford, UK). Results are displayed as the number of transcripts/ml based on an internal standard Podoplanin (PDPL). All samples were made with a known dilution series of PDPL transcript/ml against the unknown samples, and they were subjected to the same conditions. This generated a standard curve from which transcript expression of the unknown samples were calculated. This same principal applied to the expression of GAPDH to ensure sample normalisation. An iCyclerIQ thermocycler (Bio-Rad Laboratories, Hemel Hampstead, UK) was used to perform the reaction, with an optical unit that allows real-time detection of 96 reactions. The conditions were: 94° C for 5 minutes, then 100 cycles of 94° C for 10 seconds, 55° C for 35 seconds, followed by a final extension of 72° C for 10 seconds.

**Table 2:** Primers for conventional RT-PCR and real time qPCR

Gene	Primer name	Primer Sequence (5′-3′)
PEDF	SERPINF1 F50	ATCCTTTCTTCAAAGTCCCC
	SERPINF1 R50	ATTTTATGCGCAGCTTCTTC
	PEDFF1	GGTGCTACTCCTCTGCATT
	PEDFZR	**ACTGAACCTGACCGTACA**AGAAAGGATCCTCCTCCTC
GAPDH	GAPDHF8	GGCTGCTTTTAACTCTGGTA
	GAPDHR8	GACTGTGGTCATGAGTCCTT
	GAPDHF1	AAGGTCATCCATGACAACTT
	GAPDHZR1	**ACTGAACCTGACCGTACA**GCCATCCACAGTCTTCTG
PDPL	PDPLF8	GAATCATCGTTGTGGTTATG
	PDPLZR	**ACTGAACCTGACCGTACA**CTTTCATTTGCCTATCACAT

ACTGAACCTGACCGTACA represents the Z sequence.

### Immunohistochemical staining of tissues

Immunohistochemistry was performed using Vector ABC kit (Vector Laboratories, Burlingame, California, USA) as described previously [39]. The cryosections were air-dried and fixed in acetone prior to rehydration with Tris-buffered saline buffer. Incubation with a blocking reagent (10 mls of Tris-buffered saline buffer with 0.1% bovine serum albumin and 10% horse serum) was performed for 1 hour in a humidified box, followed by incubation with the primary antibody to PEDF (1:50 dilution) for a further hour. Following washing, sections were incubated with the ABC biotinylated secondary antibody for 30 minutes. Washing was repeated with further incubation of the ABC reagent for 30 minutes. ABC reagent used consisted of 5 ml of blocking reagent with 100 µl of reagent A and 100 µl of reagent B (Cat no. PK-6200; Vectastain Universal Elite ABC Kit, Vector Laboratories, Burlingame, California, USA). Washing was once again repeated, and sections were subsequently developed in DAB substrate for 5 minutes and then counterstained with Hematoxylin Gill’s Formula (Vector Laboratories, Burlingame, California, USA) for 2 minutes followed by dehydration in ethanol, clearing in xylene and mounting in DPX. The samples were visualised using a Leica DM 1000 LED microscope (Leica Microsystems Ltd, Milton Keynes, UK) at ×100 magnification. Software used to capture images was LAS EZ (Leica Application Suite, Milton Keynes, UK). All specimens were analysed anonymously. Details of demographical and clinicopathological associations were provided during experimental data analysis.

### *In vitro* cell growth assay

The standard technique used has been previously described [40]. Cells were seeded into 96-well plates at a seeding density of 3 × 10^3^ cells per well. Cell growth was measured after 1,3 and 5 days. Crystal violet cell staining was performed and analysis of absorbance was performed at 540 nm using a spectrophotometer (BioTek™ EL × 800™; BioTek).

### *In vitro* tumour cell matrigel adhesion assay

The standard technique used has been previously described [41]. Cells were seeded into Matrigel-coated (BD Matrigel™ Basement Membrane Matrix; BD Bioscience, Oxford, UK) wells (5 µg/well) at a seeding density of 4.5 × 10^4^ cells per well. Following 45 minutes of incubation, non-adherent cells were washed off using balanced salt solution buffer. The remaining cells were fixed, stained with Crystal violet and then analysed using a spectrophotometer at 540 nm.

### *In vitro* scratch migration assay

As previously described [42], cells were seeded into a 24-well plate at a seeding density of 3 × 10^5^ cells per well, and were incubated for 24 hours. After incubation, a straight-line scratch was made to the cellular monolayer using the end of a sharpened 200 µl pipette tip down the midpoint of each well. The medium was then removed from the plates carefully and replaced with 500 µl of either treatment or control. The analysis was performed using EVOS cell imaging system (Life Technologies, Paisley, UK) and images were captured every 60 minutes for a total of 6 hours. Migration distances were measured using Image-J software (National Institutes of Health, USA).

### *In vitro* cellular invasion assay

The methods used to determine the invasive ability of the cells in this study have been previously described [43]. Transwell inserts with 8 µm pores were coated with 50 µg of Matrigel^™^ and air-dried. After rehydrated, 3 × 10^4^ cells were seeded per well. Following 72 hours, the cells that migrated through the matrix to the underside of the insert were fixed and stained. The dye was solubilised using acetic acid and analysed using a spectrophotometer at 540 nm with absorbance levels as a marker of cell density presented.

### Statistical analysis

Statistical analysis was performed using Sigma plot 11.0 statistical software (Systat Software Inc.). Student *T*-test was used for parametric data and Mann–Whitney *U* test for non-parametric data, where two variables were present. The significance of associations between PEDF mRNA levels and clinicopathological variables and for functional cellular assays (except migration assays) were assessed by Kruskal–Wallis one-way analysis of variance on ranks test, and pairwise multiple comparison procedure (Dunn’s method). Two-way analysis of variance was performed for migration assay data. Parametric data were presented as mean values with error bars depicting standard error of the mean (SEM). Non-parametric data was presented, were possible as boxplots, with median values and interquartile range (IQR), with error bars depicting 95% confidence intervals. Differences were considered to be statistically significant at *p* ≤ 0.05.

## Abbreviations

cDNA: complementary deoxyribonucleic acid
EPC1: early population double level cDNA-1
GAPDH: glyceraldehyde 3-phosphate dehydrogenase
IHC: immunohistochemistry
IQR: interquartile range
mRNA: messenger RNA
PDPL: Podoplanin (internal standard)
PEDF: pigment epithelium-derived factor
qPCR: quantitative polymerase chain reaction
rhPEDF: recombinant PEDF
RNA: ribonucleic acid
SEM: standard error of the mean
TNM: tumour, node, metastases staging
VEGF: vascular endothelial growth factor
RT-PCR: reverse transcription polymerase chain reaction
